# Estimating inflammatory risk in atherosclerotic cardiovascular disease: plaque over plasma?

**DOI:** 10.1093/ehjci/jeae314

**Published:** 2024-12-07

**Authors:** Maxim E Annink, Jordan M Kraaijenhof, Cheyenne Y Y Beverloo, Reindert F Oostveen, Hein J Verberne, Erik S G Stroes, Nick S Nurmohamed

**Affiliations:** Department of Vascular Medicine, Amsterdam UMC, University of Amsterdam, Meibergdreef 9, 1105AZ Amsterdam, The Netherlands; Department of Vascular Medicine, Amsterdam UMC, University of Amsterdam, Meibergdreef 9, 1105AZ Amsterdam, The Netherlands; Department of Vascular Medicine, Amsterdam UMC, University of Amsterdam, Meibergdreef 9, 1105AZ Amsterdam, The Netherlands; Department of Vascular Medicine, Amsterdam UMC, University of Amsterdam, Meibergdreef 9, 1105AZ Amsterdam, The Netherlands; Department of Radiology & Nuclear Medicine, Amsterdam UMC, University of Amsterdam, Meibergdreef 9, 1105AZ Amsterdam, The Netherlands; Department of Vascular Medicine, Amsterdam UMC, University of Amsterdam, Meibergdreef 9, 1105AZ Amsterdam, The Netherlands; Department of Vascular Medicine, Amsterdam UMC, University of Amsterdam, Meibergdreef 9, 1105AZ Amsterdam, The Netherlands; Department of Cardiology, Amsterdam UMC, Vrije Universiteit Amsterdam, De Boelelaan 1117, 1081HV Amsterdam, The Netherlands

**Keywords:** atherosclerosis, computed tomography, imaging, inflammation, positron emission tomography

## Abstract

Inflammation is an important driver of disease in the context of atherosclerosis, and several landmark trials have shown that targeting inflammatory pathways can reduce cardiovascular event rates. However, the high cost and potentially serious adverse effects of anti-inflammatory therapies necessitate more precise patient selection. Traditional biomarkers of inflammation, such as high-sensitivity C-reactive protein, show an association with cardiovascular risk on a population level but do not have specificity for local plaque inflammation. Nowadays, advancements in non-invasive imaging of the vasculature enable direct assessment of vascular inflammation. Positron emission tomography (PET) tracers such as ^18^F-fluorodeoxyglucose enable detection of metabolic activity of inflammatory cells but are limited by low specificity and myocardial spillover effects. ^18^F-sodium fluoride is a tracer that identifies active micro-calcification in plaques, indicating vulnerable plaques. Gallium-68 DOTATATE targets pro-inflammatory macrophages by binding to somatostatin receptors, which enhances specificity for plaque inflammation. Coronary computed tomography angiography (CCTA) provides high-resolution images of coronary arteries, identifying high-risk plaque features. Measuring pericoronary adipose tissue attenuation on CCTA represents a novel marker of vascular inflammation. This review examines both established and emerging methods for assessing atherosclerosis-related inflammation, emphasizing the role of advanced imaging in refining risk stratification and guiding personalized therapies. Integrating these imaging modalities with measurements of systemic and molecular biomarkers could shift atherosclerotic cardiovascular disease management towards a more personalized approach.

## Graphical Abstract

**Graphical Abstract:**
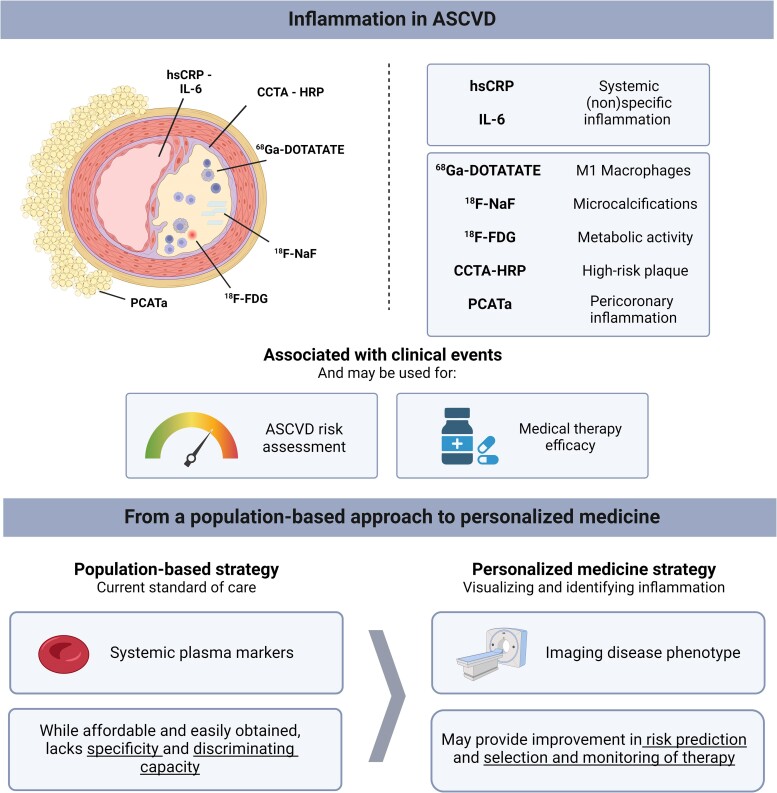
Estimating inflammatory risk: from population-based plasma markers to personalized medicine. ASCVD, atherosclerotic cardiovascular disease; hsCRP, high-sensitivity C-reactive protein; IL-6, interleukin 6; ^18^F-FDG, 18F-fluorodeoxyglucose; ^68^Ga-DOTATATE, gallium-68 DOTATATE; ^18^F-NaF, 18F-sodium fluoride; PCATa, pericoronary adipose tissue attenuation; CCTA–HRP, coronary computed tomography angiography–high-risk plaque.

## Background

Inflammation plays a critical role in the development and progression of atherosclerotic cardiovascular disease (ASCVD).^1^ Recent landmark studies investigating various anti-inflammatory therapies targeting residual inflammatory risk in ASCVD have shown mixed results regarding efficacy and safety (*Table 1*). Therefore, novel strategies are needed to precisely identify patients for whom inflammation is a residual driver of disease, allowing for targeted treatment. Traditional markers of systemic inflammation, such as high-sensitivity C-reactive protein (hsCRP), are prognostic of incident ASCVD on the population level but appear to have limited predictive power for individual risk due to their lack of specificity and direct causal association with ASCVD.^5–9^ Due to their non-specific nature, systemic biomarkers may not accurately reflect the complex local inflammatory processes that lead to plaque rupture.^5,6^ In recent years, advances in research into non-invasive imaging techniques directly assessing plaque inflammation have allowed a more precise evaluation of the inflammatory burden in ASCVD.^7^

**Figure 1:**
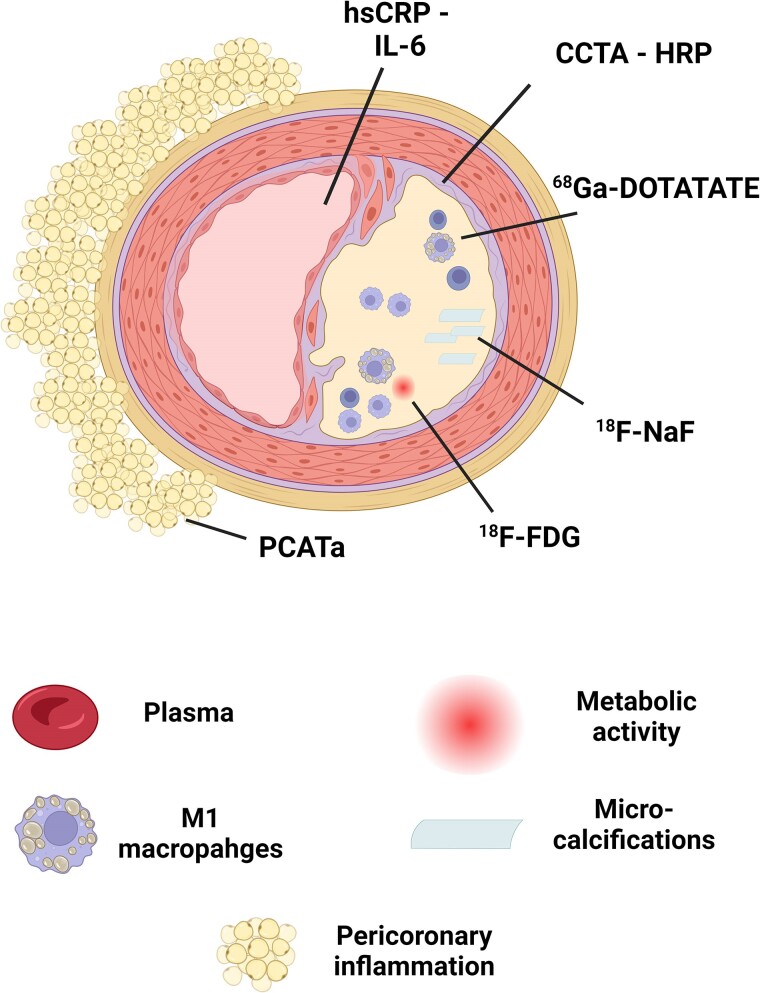
Different techniques to estimate (plaque) inflammation. Systemic inflammation markers include hsCRP (high-sensitivity C-reactive protein) and IL-6 (interleukin-6). Imaging-based inflammation assessments include ^18^F-FDG (18F-fluorodeoxyglucose) for glucose uptake, ^68^Ga-DOTATATE (Gallium-68 DOTATATE) for M1 macrophages, and ^18^FNaF (18F-Sodium Fluoride) for detecting microcalcification and active calcification within plaques. PCATa (Pericoronary Adipose Tissue attenuation) and CCTA-HRP (Coronary Computed Tomography Angiography – High-Risk Plaque) provide structural and compositional plaque evaluations.

**Table 1:** Examples of anti-inflammatory drug therapies investigated for reducing residual inflammatory risk in ASCVD

Drug	Mechanism of action	Study name	Key findings	References
Canakinumab	Monoclonal antibody inhibiting IL-1β	Canakinumab Anti-Inflammatory Thrombosis Outcomes Study (CANTOS)	At 150 mg every 3 months, significantly reduced recurrent cardiovascular events in patients with prior MI and elevated hsCRP; increased risk of fatal infections; no effect on all-cause mortality	Ridker *et al.*^2^
Colchicine	Wide-ranging anti-inflammatory effects	Low-Dose Colchicine 2 (LoDoCo2)	Low-dose colchicine (0.5 mg daily) significantly reduced cardiovascular events in patients with chronic coronary disease; higher incidence of non-cardiovascular deaths observed	Nidorf *et al.*^3^
Methotrexate	Anti-inflammatory agent, antimetabolite	Cardiovascular Inflammation Reduction Trial (CIRT)	Did not reduce inflammatory markers or cardiovascular events; associated with adverse effects including elevated liver enzymes and increased non-basal cell skin cancers	Ridker *et al.*^4^

This review examines these current and emerging methods for assessing residual inflammatory risk in atherosclerosis, focusing on systemic biomarkers and emphasizing the potential of imaging modalities to improve risk stratification and guide tailored therapeutic approaches.

## Inflammatory risk in atherosclerosis

Systemic inflammation, driven by metabolic diseases and risk factors such as obesity, chronic kidney disease, rheumatoid arthritis, and smoking, accelerates plaque progression and increases clinical event rates in ASCVD.^7–9^ A key player in this inflammatory process is the NLRP3 inflammasome, an intracellular protein complex within the inmate immune system. Upon detecting damage-associated molecular patterns or pathogen-associated molecular patterns—such as cholesterol crystals in atherosclerotic plaques—the NLRP3 inflammasome is activated.^10^ This activation leads to the cleavage of pro-caspase-1 into active caspase-1, which subsequently cleaves the inactive precursors of interleukin (IL)-1β and IL-18 into their active forms.^11^ IL-1β enhances endothelial activation by increasing adhesion molecule expression, such as intercellular adhesion molecule 1 and vascular cell adhesion protein 1, thereby promoting monocyte adhesion to plaque endothelial cells.^12^ Additionally, IL-1β stimulates the production of IL-6 by various cells, including macrophages and vascular smooth muscle cells.^11^ Elevated IL-6 levels stimulate hepatocytes to drive acute-phase reactant synthesis, most notably hsCRP. Thus, activation of the NLRP3 inflammasome triggers an inflammatory cascade that culminates in increased plasma IL-6 and hsCRP levels, both associated with higher ASCVD event rates.^13,14^ Currently, hsCRP is the primary plasma biomarker used clinically to identify systemic inflammation (*Figure 1*).

### High-sensitivity C-reactive protein

Large-scale trials over recent decades have consistently established a link between elevated levels of hsCRP and IL-6 and increased cardiovascular risk (*Table 2*). The Physician’s Health Study, a prospective cohort of healthy male physicians, found that individuals with higher hsCRP levels had an approximately three-fold increased risk of future myocardial infarction (MI) compared with those with lower levels.^15^ These findings were later replicated in women through the Women’s Health Study, demonstrating that participants with the highest hsCRP levels had about a two-fold higher risk of cardiovascular events over an 8-year follow-up period compared with those with the lowest levels, even after adjustment for other cardiovascular risk factors.^16^ A comprehensive meta-analysis involving over 160 000 individuals further confirmed that elevated hsCRP levels are associated with an increased risk of coronary heart disease and cardiovascular mortality.^17^ The clinical utility of hsCRP was also highlighted in *post hoc* analyses of the CANTOS trial, showing that the cardiovascular benefits of IL-1β inhibition were dependent on reductions in both hsCRP and IL-6 levels.^18,19^

**Table 2:** Key studies on hsCRP, IL-6, and cardiovascular risk

Study name	Population	Biomarker	Findings	References
Physician’s Health Study	1086 healthy male physicians (543 cases with vascular events, 543 controls) followed over 8 years.	hsCRP	Higher baseline hsCRP levels were associated with increased risk of MI and stroke; men in the highest CRP quartile had a three-fold higher risk of MI; aspirin reduced MI risk significantly among men with high CRP levels	Ridker *et al.*^15^
Women’s Health Study	27 939 apparently healthy women followed for a mean of 8 years	hsCRP	Higher hsCRP levels were associated with increased cardiovascular risk; women in the highest quintile had over a two-fold higher risk of cardiovascular events; hsCRP was a stronger predictor than LDL cholesterol	Ridker *et al.*^16^
Emerging Risk Factor Collaboration Meta-analysis	Meta-analysis of 160 309 individuals without prior vascular disease from 54 prospective studies	hsCRP	Each SD increase in log hsCRP (tripling of CRP levels) was associated with a 1.37-fold increase in risk of coronary heart disease and a 1.55-fold increase in cardiovascular mortality; associations were independent of conventional risk factors	Kaptoge *et al.*^17^
CANTOS *Post hoc* Analysis	10 061 patients with prior MI and elevated hsCRP (≥2 mg/L) in the CANTOS trial	hsCRP, IL-6	Cardiovascular benefits of IL-1β inhibition with canakinumab were dependent on reductions in hsCRP and IL-6; patients achieving on-treatment hsCRP < 2 mg/L or IL-6 < 1.65 ng/L had significant reductions in MACE and mortality rates	Ridker *et al.*^18,19^
CIRT Study	4786 patients with stable atherosclerosis, prior MI, or multi-vessel disease, plus diabetes or metabolic syndrome	IL-6	IL-6 levels were strongly associated with future cardiovascular events and all-cause mortality; IL-6 was a stronger predictor than hsCRP; highest risks were observed among those with elevated IL-6 and LDL cholesterol levels	Khan *et al.*^20^
MESA Study	6614 ethnically diverse, asymptomatic participants followed for a median of 14 years	IL-6	IL-6 levels were more strongly associated with future cardiovascular events, heart failure, and mortality than hsCRP; IL-6 improved risk prediction beyond traditional factors, whereas hsCRP did not after adjusting for IL-6	Ridker *et al.*^2^

Despite this robust association on a population level, evidence suggests that measuring hsCRP may have limited additional value in predicting risk of individual patients in primary prevention.^4,6^ This limitation may be partly due to the non-specific nature of CRP, which can be elevated in various inflammatory conditions beyond atherosclerosis.^21^ Moreover, Mendelian randomization studies have demonstrated that plasma hsCRP levels are not causally related to ASCVD: while genetically predicted CRP correlates with plasma hsCRP levels, it is not associated with ASCVD events.^22–24^

### Interleukin-6

IL-6, produced primarily by immune cells, contributes to atherogenesis through stimulation of monocyte recruitment and endothelial activation. Large studies have demonstrated associations between circulating levels of IL-6 and risk of ASCVD events, independent of traditional ASCVD risk factors.^25,26^ A meta-analysis confirmed that higher plasma IL-6 levels are significantly linked to a greater risk of future non-fatal MI or fatal coronary heart disease. Mendelian randomization studies have further underscored the causal role of IL-6 in ASCVD.^27–29^

Although IL-6 and hsCRP are both downstream components of NLRP3 inflammasome activation, it is crucial to note that IL-6 and hsCRP are distinct entities, with a moderate correlation between the two markers ranging from approximately *ρ* = 0.4 to 0.5.^30^ Studies like CIRT and MESA have revealed a stronger association between IL-6 and future ASCVD events than hsCRP, suggesting that IL-6 might be a more direct risk factor for ASCVD.^20,30^ Despite its potential as a more direct and causally related estimation of cardiovascular risk compared with hsCRP, widespread adoption of IL-6 measurement is hindered by challenges such as low plasma concentrations and the lack of standardized measurement techniques. Another downstream effector and marker of NLRP3 inflammasome signaling is IL-1β, which was targeted in the CANTOS trial using canakinumab.^2^ However, IL-1β’s low baseline levels in circulation limit its use as a reliable biomarker in large-scale cardiovascular risk prediction.

### Other inflammatory markers

The inflammatory response in atherosclerosis involves multiple pro-inflammatory pathways beyond the NLRP3 inflammasome. This includes the recruitment of lipoproteins and immune cells to the sub-endothelial space, which triggers a complex vascular inflammatory response involving monocytes, macrophages, neutrophils, and T-cells, all targeted in various therapeutic approaches.^31,32^ Additionally, immune regulators such as antibodies, cytokines, and chemokines play critical roles in sustaining the inflammatory cascade.^33^

Preclinical studies have identified several markers important for predicting atherosclerotic progression.^34^ While many of these markers correlate with future cardiovascular events in humans^35^ and represent potential therapeutic targets, their predictive strength is often lower than that of IL-6 and hsCRP, which have shown stronger associations with cardiovascular outcomes.^36,37^

### Correlation of systemic and plaque inflammation

These findings raise the question of whether measuring systemic inflammation markers like hsCRP and IL-6 can effectively identify patients whose ASCVD is driven by local plaque inflammation. Histological studies comparing hsCRP levels with local plaque inflammation markers show mixed results. Some studies analysing carotid plaques post-endarterectomy found no significant correlation between plasma hsCRP levels and histological markers of plaque inflammation, such as macrophage infiltration and cytokine concentrations.^38,39^ However, a larger study involving endarterectomy patients found higher hsCRP levels associated with more macrophages, larger lipid cores, and increased neovascularization in plaques.^40^

Similar inconsistencies exist in 18F-fluorodeoxyglucose positron emission tomography (^18^F-FDG PET) imaging studies. The Progression of Early Subclinical Atherosclerosis (PESA) study showed that individuals with arterial ^18^F-FDG uptake had significantly higher hsCRP levels compared with those without inflammation on PET imaging.^41^ However, smaller studies involving carotid or coronary artery disease patients did not find significant correlations between serum hsCRP and plaque ^18^F-FDG uptake.^42,43^ Optical coherence tomography (OCT) imaging studies, which can visualize macrophages in the vessel wall, also yielded inconclusive results: a small study found higher hsCRP independently predicted macrophage presence on OCT,^44^ but a larger study did not confirm this correlation.^45^ In conclusion, studies comparing plasma inflammation with plaque inflammation suggest substantial differences between systemic and local inflammation markers.

### Limitations of systemic biomarkers

Although hsCRP is a readily accessible plasma biomarker with robust population-level associations with cardiovascular events, its individual-level predictive utility is limited. This limitation is due to hsCRP’s non-specific nature; it is not causally related to ASCVD and can be elevated in various inflammatory conditions beyond atherosclerosis. For patients with inflammation driven by pathways other than NLRP3, hsCRP may not be elevated.^32^ Other markers, such as IL-6, are likely causally linked to ASCVD but are not yet clinically applicable due to difficulties in measurement and standardization.

## Atherosclerotic plaque inflammation imaging

In order to improve the assessment of cardiovascular risk and identify those patients who stand to benefit most from targeted anti-inflammatory therapies, many advanced imaging techniques that visualize inflammation within atherosclerotic plaques have been studied. These non-invasive modalities can evaluate the presence of vascular inflammation and plaque characteristics that drive the progression of atherosclerosis and plaque rupture. In this section, we explore imaging modalities for assessing atherosclerotic plaque inflammation, highlighting their potential to improve ASCVD risk assessment.

### PET–computed tomography imaging

PET is a functional molecular imaging technique used to visualize and measure metabolic processes by displaying the concentrations and distributions of injectable radioactive tracers. To facilitate precise anatomical localization, PET is often combined with computed tomography (CT) or magnetic resonance imaging (MRI).^46^

#### ^18^F-fluorodeoxyglucose

One of the most frequently used PET tracers in vascular inflammation imaging is ^18^F-FDG, a glucose analogue.^47^ Due to their increased glucose uptake, metabolically active inflammatory cells, such as activated macrophages, can be visualized with ^18^F-FDG.^48,49^ Visualization of plaques with ^18^F-FDG enables early detection of lesions, starting in the fatty streak formation phase. *In vitro* studies have shown that stimulated macrophages transforming into foam cells exhibit higher ^18^F-FDG uptake than control macrophages and fully differentiated foam cells.^50^

Several clinical trials have assessed the ability of ^18^F-FDG PET to estimate cardiovascular risk (*Table 3*). In a subgroup analysis of the ongoing PESA study, ^18^F-FDG PET/MRI detected widespread vascular inflammation in up to 50% of a large population of middle-aged, low-risk individuals with sub-clinical atherosclerosis. Interestingly, only a small minority of plaques in these patients showed signs of increased ^18^F-FDG uptake, suggesting that ^18^F-FDG may have the capability to identify atherosclerotic lesions before they become apparent with other imaging techniques.^55^

**Table 3:** Overview of reviewed studies on imaging biomarkers in cardiovascular prognostication

Author	Methods	Population	Findings
^18^F-FDG PET			
Zheng *et al.*^51^	Prospective cohort study evaluating the association of plaque ^18^F-FDG uptake and recurrent cerebral ischaemic events and MACE (90-day follow-up)	131 patients with recent TIA or ischaemic stroke and significant ipsilateral carotid artery stenosis	^18^F-FDG uptake (per 1 g/mL SUV_max_) in the ipsilateral carotid plaque was associated with recurrent ipsilateral stroke (HR: 5.92, *P* < 0.001) ^18^F-FDG uptake (per 1 g/mL SUV_max_) in the ipsilateral carotid plaque was associated with TIA or MACE (HR: 3.33, *P* < 0.001)
McCabe *et al.*^52^	Pooled analysis of three prospective studies that evaluated the association of ipsilateral carotid ^18^F-FDG uptake and recurrent stroke (median 5-year follow-up)	181 patients with recent TIA or stroke and ipsilateral carotid stenosis	^18^F-FDG uptake (per 1 g/mL SUV_max_) in the ipsilateral plaque could predict 5-year ipsilateral recurrent stroke, after adjustment for common stroke risk factors (HR: 1.98; *P* = 0.02)
Chowdhury *et al.*^53^	Prospective cohort study comparing restenosis-free survival between patients with high ^18^F-FDG and low ^18^F-FDG uptake of the superficial femoral artery (12-month follow-up)	50 patients with recent angioplasty for symptomatic peripheral arterial disease	^18^F-FDG uptake could successfully discriminate between patients who developed restenosis 1 year post-angioplasty and those who did not (*P* < 0.0001; log-rank *P* < 0.001)
Kelly *et al.*^54^	Prospective cohort study evaluating the association of ipsilateral carotid ^18^F-FDG uptake and recurrent stroke (90-day follow-up)	109 patients with recent TIA or stroke and ipsilateral carotid stenosis	^18^F-FDG uptake (per 1 g/mL SUV_max_) in the ipsilateral carotid artery was associated with recurrent ipsilateral stroke (HR: 2.2, *P* < 0.001)
Fernández-Friera *et al.*^55^	Cross-sectional study using ^18^F-FDG PET/MRI to assess arterial inflammation	755 middle-aged individuals (ages 40–54; 83.7% men) with sub-clinical atherosclerosis	Arterial ^18^F-FDG uptake was observed in 48.2% of participants ^18^F-FDG uptake was associated with higher plaque burden, and increased uptakes were more common in participants with risk factors (*P* < 0.01) Most uptake occurred in arterial segments without detectable plaques (61.5%), and there was a weak but positive correlation between the number of plaques and ^18^F-FDG uptake (*ρ* = 0.25; *P* < 0.001), indicating that inflammation may play a role in early atherosclerosis
Tarkin *et al.*^56^	Prospective study comparing ^18^F-FDG PET and 68Ga-DOTATATE PET for imaging atherosclerotic inflammation	42 patients with atherosclerosis	^18^F-FDG uptake successfully differentiated culprit from non-culprit carotid lesions (median difference: 0.12; IQR: 0.0–0.23; *P* = 0.008) and high-risk from lower-risk coronary arteries (ROC AUC: 0.76; 95% CI: 0.62–0.91; *P* = 0.002) 64% of patients had uninterpretable coronary ^18^F-FDG scans due to myocardial spillover
Moon *et al.*^57^	Retrospective study evaluating the predictive power of measuring carotid vascular inflammation by ^18^F-FDG PET/CT for MACE (average follow-up of 4.2 years)	1089 asymptomatic adults	High carotid ^18^F-FDG uptake was predictive of MACE (HR: 2.98; CI: 1.17–7.62; *P* = 0.022) No statistically significant improvement of net reclassification index by adding ^18^F-FDG uptake to FRS
Kim *et al.*^58^	Prospective study examining the association between ^18^F-FDG PET imaging of carotid inflammation and early recurrent ischaemic lesions	21 patients with symptomatic carotid atherosclerosis	Patients with recurrent lesions showed higher ^18^F-FDG uptake in carotid plaques (SUV_max_ 3.07 ± 0.79) compared with those without (SUV_max_ 2.17 ± 0.68; *P* = 0.013)
Figueroa *et al.*^59^	Retrospective study evaluating the predictive power of measuring aortic vascular inflammation by ^18^F-FDG PET/CT for MACE (median follow-up of 4.2 years)	513 asymptomatic adults	^18^F-FDG uptake in the ascending aorta strongly predicted the incidence of MACE (HR: 4.71, *P* < 0.001) Addition of aortic ^18^F-FDG uptake to the FRS led to statistically significant net reclassification improvements for CVD risk estimation
^18^F-NaF PET			
Bhakta *et al.*^60^	*Post hoc* analysis of a study using ^18^F-NaF PET to assess carotid plaque micro-calcification and its association with recurrent neurovascular events	18 individuals with ischaemic stroke due to ipsilateral carotid stenosis	Higher ^18^F-NaF uptake in symptomatic carotid plaques at baseline was associated with increased risk of recurrent ipsilateral ischaemic stroke or TIA over 6 months (adjusted OR: 1.24; 95% CI: 1.03–1.50)
Daghem *et al.*^61^	Prospective study assessing changes in coronary ^18^F-NaF over 12 months	111 patients with established coronary artery disease	Baseline coronary ^18^F-NaF uptake was associated with higher calcium scores [294 AU (IQR 116–483 AU) vs. 72 AU (IQR 8–222 AU); *P* < 0.001] and faster progression of calcification [39 AU (IQR 10–82 AU) vs. 12 AU (IQR 1–36 AU); *P* < 0.001]
Moss *et al.*^62^	Prospective, longitudinal cohort study assessing coronary atherosclerotic plaque activity using ^18^F-NaF PET CT	995 patients with a history of recent myocardial infarction and multi-vessel coronary artery disease	No significant association was found between increased plaque activity and the primary outcome of cardiac death, non-fatal myocardial infarction, or unplanned revascularization (HR, 1.25; 95% CI, 0.89–1.76; *P* = 0.2) Higher plaque activity was linked to an increased risk of cardiac death or non-fatal myocardial infarction (HR, 1.82; 95% CI, 1.07–3.10; *P* = 0.03) and overall mortality (HR, 2.43; 95% CI, 1.15–5.12; *P* = 0.02)
Doris *et al.*^63^	Prospective study of the association between coronary ^18^F-NaF uptake and progression of coronary calcification as measured by CT calcium scoring (12-month follow-up)	183 patients with multi-vessel coronary atherosclerosis	Progression of overall calcium score was significantly increased between patients with high and low ^18^F-NaF uptake at baseline (97 vs. 35 AU; *P* < 0.0001), which was fully driven by increased calcification in sites of increased ^18^F-NaF uptake Coronary ^18^F-NaF uptake correlated with the progression of calcium score (*ρ* = 0.37; *P* < 0001)
Kwiecinski *et al.*^64^	*Post hoc* analysis of several prospective observational studies, studying the association between coronary ^18^F-NaF uptake and subsequent fatal or non-fatal myocardial infarction (median 42-month follow-up)	293 patients with known coronary artery disease	Myocardial infarction only occurred in those with increased coronary ^18^F-NaF uptake Use of ^18^F-NaF outperformed coronary calcium scoring and clinical risk scores for prediction of myocardial infarction on receiver operator curve analysis
Chowdhury *et al.*^53^	Prospective observational cohort study evaluating ^18^F-NaF PET/CT as a predictor of restenosis following PTA	50 patients with symptomatic peripheral arterial disease	Baseline ^18^F-NaF uptake in the femoral arteries was significantly higher in those who developed restenosis at 12 months [TBR 2.61 (IQR 2.50–2.77) vs. 1.69 (IQR 1.54–1.77); *P* < 0.001] A cut-off TBR_max_ value of 2.11 for ^18^F-NaF uptake showed strong predictive value for identifying patients at higher risk of restenosis (log-rank *P* < 0.001)
Joshi *et al.*^65^	Prospective study to comparing ^18^F-NaF uptake of culprit and non-culprit coronary plaques with other imaging modalities	40 patients with MI and 40 patients stable angina who underwent ^18^F-NaF and ^18^F-FDG PET–CT, and invasive coronary angiography	In 37 (93%) MI patients, the highest coronary ^18^F-NaF uptake was seen in the culprit plaque [median maximum tissue-to-background ratio: culprit 1.66 (IQR 1.40–2.25) vs. highest non-culprit 1.24 (1.06–1.38), *P* < 0.0001] ^18^F-NaF uptake was observed at all sites of carotid plaque ruptures, correlating with histological features of ongoing calcification, macrophage invasion, apoptosis and necrosis
^68^Ga-DOTATATE PET			
Oostveen *et al.*^66^	Prospective study evaluating the effect of atorvastatin treatment on ^68^Ga-DOTATATE uptake across the cardio-hematopoietic axis	22 individuals with Type 2 diabetes	Atorvastatin treatment resulted in a 31% lower coronary TBR_max_ (95% CI −50 to −12). Uptake in bone marrow and spleen was also significantly reduced, with a mean percentage reduction of −15% (95% CI −27 to −4) and −17% (95% CI −32 to −2), respectively
Tarkin *et al.*^56^	Cross-sectional study comparing detection of atherosclerotic inflammation between ^68^Ga-DOTATATE and ^18^F-FDG PET/CT imaging	42 patients with atherosclerosis	Compared with ^18^F-FDG, ^68^Ga-DOTATATE offered superior coronary imaging and better power to discriminate high- from low-risk coronary lesions
Mojtahedi *et al.*^67^	*Post hoc* analysis assessing coronary ^68^Ga-DOTATATE uptake in vulnerable atherosclerotic plaques and fibrotic plaques	44 neuroendocrine tumour patients	Compared with normal coronary arteries, there was significantly increased ^68^Ga-DOTATATE uptake in vulnerable atherosclerotic and fibrotic coronary plaques
Li *et al.*^68^	Murine study to assess co-localization of SSTR2 and macrophages in plaque	ApoE^−/−^ mice on high-cholesterol diet	^68^Ga-DOTATATE co-localized with macrophage-rich plaques
Li *et al.*^69^	*Post hoc* analysis evaluating the correlation between ^68^Ga-DOTATATE uptake in large arteries and ^18^F-FDG uptake	16 neuroendocrine tumour patients	^68^Ga-DOTATATE uptake within large arteries correlated significantly with ^18^F-FDG uptake, calcium presence, and hypertension
Rominger *et al.*^70^	*Post hoc* analysis evaluating the correlation between ^68^Ga-DOTATATE uptake in the LAD and coronary calcium burden	70 neuroendocrine tumour patients	The LAD TBR_max_ was significantly correlated with the presence of calcified plaques (*R* = 0.34; *P* < 0.01), prior vascular events (*R* = 0.26; *P* < 0.05), and male sex (*R* = 0.29; *P* < 0.05)
CCTA High-risk plaque			
Chang *et al.*^71^	Nested case-control study within 25 251 patients undergoing CCTA; analysed plaque characteristics and HRPs	234 ACS patients and 234 matched controls	% stenosis, plaque burden, fibro-fatty and necrotic core volumes, and HRPs were independently associated with increased ACS risk (*P* < 0.05) Over 65% of ACS patients had non-obstructive CAD; 52% had HRPs
Ferencik *et al.*^72^	Nested observational cohort study within the PROMISE trial; assessed high-risk plaque on CCTA	4415 stable outpatients with chest pain	High-risk plaque present in 15.3%; significant stenosis in 6.3% High-risk plaque associated with higher MACE rate (6.4 vs. 2.4%; HR 2.73; *P* < 0.001) Association remained after adjusting for ASCVD risk score and significant stenosis (adjusted HR 1.72; *P* = 0.01)
Tzolos *et al.*^73^	*Post hoc* analysis of the SCOT-HEART trial; assessed LAP burden via CCTA	1697 participants	LAP burden >4% strongly predicted future myocardial infarction (HR: 4.87; *P* < 0.0001) LAP burden showed good predictive accuracy (AUC = 0.71; 95% CI: 0.62–0.80)
Motoyama *et al.*^74^	Prospective study that evaluated HRP features via CCTA	3158 patients undergoing CCTA; mean follow-up of 3.9 years	HRP was an independent predictor of ACS [16.3% in HRP(+) vs. 1.4% in HRP(−); *P* < 0.0001]
CCTA PCATa imaging			
van Rosendael *et al.*^75^	Prospective cohort study assessing PCAT attenuation’s predictive value for long-term MACE	483 symptomatic CAD patients	PCAT attenuation did not predict MACE No significant difference in PCAT attenuation between patients with and without MACE
Chan *et al.*^76^	Prospective cohort study which evaluated the predictive value of PCATa for all-cause and cardiac mortality in two cohorts	3393 patients who underwent CCTA for a clinical indication	PCATa of each of the coronary vessels was predictive of cardiac mortality and MACE even after adjustment for classical cardiovascular risk factors
Wen *et al.*^77^	Retrospective study assessing the prognostic value of PCATa and CAD-RADS categories for MACE	1313 patients with acute chest pain undergoing CCTA	RCA PCATa was predictive of MACE (HR: 1.033; *P* = 0.006) CAD-RADS alone provided better risk stratification than PCAT attenuation alone (C-index 0.760 vs. 0.712; *P* = 0.036) Adding PCAT attenuation to CAD-RADS did not significantly improve prognostic performance for MACE (combined *C*-index: 0.777 vs. CAD-RADS alone: 0.760; *P* = 0.129)
Chatterjee *et al.*^78^	Prospective cohort study evaluating PCATa’s predictive value for adverse cardiac events	344 patients with (suspected) CAD undergoing invasive coronary angiography	PCAT attenuation values measured in the RCA, LAD, and LCx did not significantly predict MACEs (HR for RCA: 0.96, CI: 0.75–1.22; *P* = 0.71; LAD: 1.31, CI: 0.96–1.78; *P* = 0.09; and LCx: 0.98, CI: 0.78–1.22; *P* = 0.84)
Lee *et al.*^79^	Prospective cohort study examining the association between changes in PCATa and coronary plaque progression on CCTA	474 patients	An increase in PCATa was significantly associated with the progression of total plaque volume (estimate = 0.275, 95% CI: 0.004–0.545; *P* = 0.047), which was driven by the progression of fibrous plaque volume, but not by progressions of calcified plaque volume
Tzolos *et al.*^73^	*Post hoc* SCOT-HEART analysis examining the predictive value of PCATa and LAP burden for myocardial infarction	1697 participants undergoing CCTA for stable chest pain	Elevated PCATa around the right coronary artery was independently predictive of future myocardial infarction (HR: 2.45; *P* = 0.01) LAP burden alone showed a higher predictive AUC for MI compared with PCATa but combining both metrics further increased predictive accuracy compared with PCATa alone (AUC = 0.64 vs. 0.75; *P* = 0.037)
van Diemen *et al.*^80^	Prospective study evaluating the prognostic value of PCATa imaging in relation to CCTA-derived plaque volume and PET-determined myocardial ischemia for a composite endpoint of death and non-fatal myocardial infarction (5-year median follow-up)	539 patients who underwent CCTA due to suspected CAD	Elevated RCA PCATa was associated with a poorer prognosis (unadjusted HR: 2.84; *P* = 0.003) and retained prognostic value when adjusted for clinical characteristics and imaging variables (adjusted HR: 2.45; *P* = 0.011)
Bengs *et al.*^81^	Cohort study assessing the incremental prognostic value of FAI in combination with myocardial perfusion imaging and coronary artery calcium scoring	492 patients undergoing CCTA and myocardial perfusion imaging	After adjusting for coronary artery calcium scoring, PCATa was no longer a significant predictor of MACE (*P* = 0.279), indicating limited incremental prognostic value beyond CACS and MPI
Oikonomou *et al.*^82^	*Post hoc* analysis of the CRISP-CT study, investigating the relationship between PCATa and high-risk plaque features on CCTA	3912 patients undergoing CCTA	Elevated PCATa significantly increased cardiac mortality risk in both patients with high-risk plaque on CCTA (HR: 7.33, 95% CI: 3.22–16.67; *P* < 0.001) as well as without (HR: 5.65, 95% CI: 2.65–12.03; *P* < 0.001)
Goeller *et al.*^83^	Prospective cohort study evaluating the relationship between PCAT attenuation and coronary plaque burden progression on CCTA	111 patients with stable CAD	Changes in PCAT attenuation correlated with changes in non-calcified plaque burden (*r* = 0.55, *P* < 0.001) and low-density plaque burden (*r* = 0.24, *P* = 0.01), but not with calcified plaque burden (*P* = 0.3) Baseline PCAT attenuation was an independent predictor of increased non-calcified and total plaque burden at follow-up (OR: 3.07, 95% CI: 1.4–7.0; *P* < 0.008)
Oikonomou *et al.*^84^	*Post hoc* analysis of a prospective cohort study, which evaluated the predictive value of PCATa for all-cause and cardiac mortality	1872 patients in the derivation cohort and 2040 patients in the validation cohort undergoing CCTA	High perivascular PCATa around the proximal right coronary artery and left anterior descending artery predicted all-cause and cardiac mortality in both cohorts A cut-off of ≥−70.1 HU for PCATa indicated a steep increase in cardiac mortality (derivation cohort HR: 9.04; CI: 3.35–24.40; *P* < 0.0001; validation cohort HR: 5.62; CI: 2.90–10.88; *P* < 0.0001)

AUC, area under the curve; CAD, coronary artery disease; CACS, coronary artery calcium score; CAD-RADS, coronary artery disease – reporting and data system; CCTA, coronary computed tomography angiography; CVD, cardiovascular disease; FAI, fat attenuation index; HR, hazard ratio; IQR, inter-quartile range; LAD, left anterior descending artery; LCx, left circumflex artery; MPI, myocardial perfusion imaging; OR, odds ratio; RCA, right coronary artery; TBR, target-to-background ratio; TIA, transient ischemic. attack.

Large-scale studies have validated the ability of ^18^F-FDG PET to retrospectively identify individuals at risk for cardiovascular events. In one retrospective cohort study of over 1000 healthy participants from a cancer screening programme, those with higher ^18^F-FDG uptake in the carotid arteries had a significantly higher incidence of major adverse cardiovascular events (MACEs) compared with those with lower uptake.^57^ Another retrospective cohort study involving 513 healthy individuals who had undergone 18F-FDG imaging for oncological evaluation found that elevated ^18^F-FDG uptake in the ascending aorta strongly predicted the incidence of MACE over the follow-up period.^59^ In both studies, risk assessment using whole-vessel ^18^F-FDG uptake combined with the Framingham risk score (FRS), a traditional clinical risk estimation tool, outperformed the FRS alone. However, these studies are limited by their retrospective design and potential selection bias. Small prospective studies have demonstrated the predictive value of ^18^F-FDG for recurrent stroke in patients following symptomatic carotid artery disease^51,52,54,58,85^ and following recent percutaneous transluminal angioplasty (PTA) of the lower extremities.^53^ More definitive insights are expected from large, prospective, event-driven studies such as the PESA study.

Beyond diagnosing atherosclerotic disease, ^18^F-FDG PET imaging has been employed to monitor the impact of anti-inflammatory treatments in research settings. Statins, known primarily for lowering LDL cholesterol, also exhibit anti-inflammatory properties and have been assessed using ^18^F-FDG imaging.^86^ A meta-analysis encompassing 10 intervention studies with a total of 287 participants revealed a significant decrease in ^18^F-FDG uptake following statin therapy.^87^ In line with this, anti-inflammatory drugs such as losmapimod and dalcetrapib, which failed to reduce ^18^F-FDG uptake in Phase 2 studies, subsequently did not demonstrate cardiovascular benefits in large outcome studies.^88^

However, the application of ^18^F-FDG imaging in atherosclerosis is constrained by several challenges, primarily due to the tracer’s lack of specificity. Since ^18^F-FDG is a glucose analogue, most metabolically active cells—not just inflammatory cells—take up ^18^F-FDG. In the myocardium, this can result in physiological myocardial uptake, causing spillover that severely hampers the accurate assessment of coronary artery uptake. A study by Tarkin *et al.*^56^ found that a considerable proportion of their cardiac ^18^F-FDG scans were uninterpretable due to myocardial spillover. Additionally, ^18^F-FDG uptake is affected by plasma glucose levels, posing challenges in patients with diabetes mellitus.^89^ Another impracticality is caused by ^18^F-FDG uptake in skeletal muscles, which requires patients limit physical activity during the 24 h before the scan. Furthermore, it is still debatable which of the macrophage subsets (i.e. pro-inflammatory M1 or regulatory M2 macrophages) exhibit the highest ^18^F-FDG uptake.

General challenges in PET imaging include the limited spatial resolution, ranging from 5 to 8 mm, which is sub-optimal, especially considering the relatively small size of human atherosclerotic plaques.^90^ Practical factors also limit the large-scale use of PET imaging in cardiovascular risk assessment. For instance, in the current clinical landscape, PET imaging is only carried out in specialized centres. Imaging protocols are currently unstandardized, and the radiation dose of ∼1–3 mSv per 100 MBq may raise safety concerns (*Table 4*).

**Table 4:** Advantages and disadvantages of non-invasive approaches for estimating residual inflammatory risk

Imaging modality	Advantages	Disadvantages	Spatial resolution (mm)	Radiation dose (mSv/100 MBq)	Clinical preparation	Costs (€)
^18^F-FDG	Widely used in research Visualizes metabolically active inflammatory cells (e.g. activated macrophages) Enables early detection of atherosclerotic lesions Can monitor response to anti-inflammatory treatments (e.g. statins)	Radiation exposure (∼7–10 mSv) Non-specific uptake; reflects overall metabolic activity, not specific to inflammation Myocardial uptake causes spillover, hindering coronary artery imaging Affected by plasma glucose levels; challenging in diabetic patients Limited spatial resolution (5–8 mm); sub-optimal for small plaques Requires special patient preparation High cost and limited accessibility Unstandardized imaging protocols	4–5	1–3	Low-carbohydrate diet and/or fasting Limit physical activity 24 h prior	∼1000–1200
^68^Ga-DOTATATE	Specific binding to SSTR2 on pro-inflammatory M1 macrophages Improved coronary imaging over 18F-FDG; no myocardial spillover Higher specificity for plaque inflammation Potential to monitor therapeutic response	Radiation exposure Limited availability; requires PET facility with 68 Ga generator Need for standardization of imaging protocols Limited data in ASCVD; more research needed High cost	5–7^121^	2–3^122^	None	∼1000–1200
^18^F-NaF	Detects active micro-calcification in plaques (early phase of inflammation) Improved specificity for coronary artery imaging Not affected by myocardial spillover Predicts plaque progression and vulnerability Associated with future cardiovascular events in research studies	Radiation exposure Limited availability Need for standardization of imaging protocols Larger-scale, prospective studies needed for validation High cost	4–5	5–8	None	∼1000–1200
CCTA: high-risk plaque features	High-resolution imaging of coronary arteries (0.4–0.6 mm) Non-invasive assessment of plaque composition and morphology Identifies high-risk plaque features (e.g. positive remodelling, LAP, napkin-ring sign, spotty calcifications) Provides prognostic information beyond stenosis severity Widely available; performed routinely Likely cost-effective compared with PET imaging	Radiation exposure Requires iodinated contrast agents; risk of allergic reactions or nephrotoxicity Operator-dependent variability in identifying plaque features	0.4–0.6	1–3	Sub-lingual nitroglycerine Heart rate reduction with beta-blockers	∼250–300 (CCTA only)
CCTA: PCAT attenuation	Assesses coronary inflammation indirectly by measuring attenuation of PCAT High-resolution imaging Potential to improve risk stratification beyond traditional measures No additional cost or radiation when added to standard CCTA	Radiation exposure Requires contrast agents Need for standardization of imaging protocols and validation of PCAT attenuation thresholds Influenced by technical factors and patient anatomy Mixed evidence in predicting cardiovascular events; further validation needed	0.4–0.6	1–3	Sub-lingual nitroglycerine and heart rate reduction with beta-blockers	∼250–300 (CCTA only)

Advantages and disadvantages of current and future approaches for estimating (plaque) inflammation in ASCVD.

#### ^18^F-sodium fluoride

Intra-plaque calcifications smaller than 50 μm, known as micro-calcifications, are indicators of inflammation and are associated with an elevated risk of plaque rupture and subsequent atherothrombotic complications.^91^  ^18^F-sodium fluoride (^18^F-NaF), a radioactive tracer traditionally used in diagnosing metastatic bone cancer, has been investigated as a marker of active vascular micro-calcification—an early manifestation of plaque inflammation (*Table 3*).^63^

In a pivotal study involving patients with MI and stable angina, ^18^F-NaF detected culprit lesions in 93% of MI patients. Plaques with elevated ^18^F-NaF activity were also more likely to have high-risk features on ultrasonography.^65^ Other studies have associated ^18^F-NaF activity with plaque vulnerability features, including histology and pericoronary adipose tissue (PCAT) attenuation.^92,93^ While ^18^F-NaF uptake correlates with coronary artery calcifications seen on CT scans,^94^ it uniquely identifies micro-calcifications and actively calcifying areas, unlike CT scans that image larger calcium deposits.^95^ In one study, only 12% of calcified plaques identified by CT showed increased ^18^F-NaF uptake, whereas 75% exhibited elevated ^18^F-NaF uptake in areas without visible calcifications.^90^ Another study found that over 40% of patients with elevated coronary artery calcium scores did not have elevated ^18^F-NaF uptake, suggesting that ^18^F-NaF distinguishes between active atherosclerosis and advanced but inactive plaques.^96^ Several studies have demonstrated that ^18^F-NaF uptake in the coronary arteries is associated with the progression of plaque calcification on coronary CT angiography (CCTA).^61,63^ Notably, the calcium score progressed exclusively in segments of the coronary vasculature with ^18^F-NaF uptake while remaining stable in segments without it.

The potential of ^18^F-NaF in predicting incident cardiovascular events has been demonstrated in several studies. Recent prospective studies in patients with established ASCVD have shown that coronary ^18^F-NaF uptake is associated with both all-cause mortality and a composite endpoint of cardiac death and non-fatal MI,^62^ surpassing the predictive accuracy of coronary calcium scoring and other established risk indices.^64^ Additionally, carotid plaque uptake of ^18^F-NaF has been shown to predict recurrent ipsilateral ischaemic stroke.^60^ In patients undergoing lower limb PTA, ^18^F-NaF uptake in the femoral arteries was associated with incident restenosis at 1-year follow-up.^53^

Comparative studies of ^18^F-FDG and ^18^F-NaF uptake in coronary arteries have consistently demonstrated that ^18^F-NaF uptake has a stronger correlation with culprit lesion sites and cardiovascular outcomes. This superiority is likely due to myocardial spillover in ^18^F-FDG imaging. In a study by Joshi *et al.*,^65^  ^18^F-FDG uptake was often obscured by myocardial uptake and did not show a significant difference between culprit and non-culprit lesions, in contrast to ^18^F-NaF uptake. Other studies found that ^18^F-NaF uptake in the coronary arteries and aorta correlated significantly with 10-year Framingham Risk Scores and a history of revascularization and MACE, whereas ^18^F-FDG did not show such correlation.^96,97^

Moreover, a recent study demonstrated the reversibility of ^18^F-NaF uptake; participants with sub-clinical atherosclerosis showed a significant reduction in ^18^F-NaF uptake after 6 months of rosuvastatin treatment.^98^ The primary limitation of using ^18^F-NaF is exposure to ionizing radiation. While it offers enhanced specificity compared with ^18^F-FDG in imaging coronary inflammation, larger-scale studies are still needed to comprehensively evaluate its effectiveness in predicting cardiovascular events (*Table 3*).

#### Gallium-68 DOTATATE

An emerging tracer of interest is gallium-68-labelled [1,4,7,10-tetraazacyclododecane-N,N′,N″,N″′-tetra-acetic acid]-D-Phe1, Tyr3-octreotate [gallium-68 DOTATATE (^68^Ga-DOTATATE)], a somatostatin analogue extensively used for assessing neuroendocrine tumours. Its efficacy in visualizing plaque inflammation stems from its binding to somatostatin receptor Subtype 2 (SSTR2) on pro-inflammatory M1 macrophages.^56,68^ Macrophages are principal immune cells in atherosclerosis, comprising both resident macrophages and monocytes that migrate into the vessel wall upon chemotactic stimulation, where they may undergo local proliferation.^99^ Notably, in human lesions, pro-inflammatory M1 macrophages are predominantly located in rupture-prone areas, such as the lesion shoulder and fibrous cap, whereas anti-inflammatory M2 macrophages are primarily found in regions less susceptible to rupture, such as the adventitia.^100^

Uptake of ^68^Ga-DOTATATE in the coronary vessel wall has been linked to the presence of calcified atherosclerotic plaques and various cardiovascular risk factors (*Table 3*).^67,69,70^ In a prospective study involving patients with different degrees of atherosclerotic disease, ^68^Ga-DOTATATE PET/CT imaging demonstrated high macrophage specificity and improved discrimination compared with ^18^F-FDG PET/CT, particularly in distinguishing between culprit and non-culprit lesions in coronary and carotid arteries.^56^ The specific binding to M1 macrophages of ^68^Ga-DOTATATE resulted in no myocardial spillover, whereas myocardial spillover in ^18^F-FDG scans rendered a large majority of the scans uninterpretable.

In patients with type 2 diabetes, a significant reduction in ^68^Ga-DOTATATE uptake was observed following 3 months of statin treatment,^66^ indicating its potential utility in monitoring therapeutic response. Conversely, a smaller study involving patients with symptomatic carotid atherosclerotic plaques did not find significant differences in ^68^Ga-DOTATATE uptake between symptomatic and asymptomatic plaques.^101^ This discrepancy was attributed to the histologically confirmed absence of SSTR2 expression in the carotid arteries of these patients. Consequently, additional larger prospective studies are required to validate its role in plaque identification and risk stratification (*Table 3*). As with the other PET tracers discussed in this review, radiation exposure is an important barrier to the widespread implementation of ^68^Ga-DOTATATE PET in atherosclerosis (*Table 4*).

### Coronary CT angiography

CCTA offers high-resolution, 3D imaging of the coronary arteries. In current clinical practice, CCTA is primarily used for the assessment of luminal stenosis and the evaluation of the overall burden of coronary artery atherosclerosis. The extent and severity of stenoses detected by CCTA serve as strong predictors of MACE, providing incremental prognostic value for MI and mortality beyond traditional clinical risk factors.^102^

Beyond evaluating luminal stenosis, CCTA can provide detailed insights into plaque composition and morphology. As will be discussed, high-risk plaque features identifiable on CCTA—including positive remodelling, low-attenuation plaque (LAP), the napkin-ring sign, and spotty calcifications—have been closely linked to increased plaque vulnerability and adverse cardiovascular events (*Table 3*).^103,104^ Positive remodelling refers to the outward expansion of the vessel wall at the site of a plaque, potentially concealing significant plaque burden and reflecting active inflammation within the arterial wall. LAP is characterized by regions with very low Hounsfield units (HUs, typically below 30 HU), indicating a lipid-rich necrotic core—a hallmark of vulnerable plaques prone to rupture.^105^ The napkin-ring sign appears as a ring-like attenuation pattern surrounding a low-attenuation core on CCTA images, signifying a thin-cap fibroatheroma with a high risk of rupture. Spotty calcifications are small, punctate calcium deposits within plaques, associated with increased plaque instability and inflammatory activity.^106^

Several landmark studies have demonstrated the prognostic significance of these high-risk plaque features. In a cohort of over 3000 patients undergoing CCTA, Motoyama *et al.*^74^ found that patients with high-risk plaques had a significantly higher incidence of acute coronary syndrome (ACS) compared with those without high-risk plaques. The ICONIC study matched patients who developed ACS after baseline CCTA with those who did not, finding that the presence of LAP and other high-risk features was higher among those who experienced ACS.^71^ These features predicted future ACS events independently of traditional risk factors and stenosis severity. Similarly, the PROMISE trial showed that high-risk plaque features identified on CCTA were associated with an increased risk of MACE, especially in patients without obstructive coronary artery disease, underscoring the incremental value of detailed plaque characterization.^72^ The SCOT-HEART trial further reinforced the importance of these features by demonstrating that LAP burden was independently associated with an increased risk of MI, even after adjusting for total plaque burden, coronary artery calcium score, and the presence of obstructive coronary artery disease.^73^ This underscores that specific plaque characteristics can provide prognostic information beyond traditional assessments of stenosis severity and overall plaque burden.

Importantly, the presence of these high-risk plaque features is intimately linked to underlying inflammatory processes within atherosclerotic lesions. Inflammation plays a pivotal role in plaque destabilization and progression, leading to morphological changes detectable by CCTA. For example, LAP has been associated with heightened inflammatory activity as measured by molecular imaging techniques such as ^68^Ga-DOTATATE and ^18^F-FDG PET,^56^ as well as plaque micro-calcification detected by ^18^F-NaF PET.^107^ These findings indicate that regions identified as LAP on CCTA correspond to areas of active inflammation and metabolic activity at the molecular level.

Recently, assessing PCAT attenuation (PCATa) using CCTA has emerged as a novel approach to quantifying vascular inflammation (*Table 3*). PCAT surrounds the coronary arteries and consists of adipocytes and various immune cell types, playing a significant role in modulating the inflammatory environment of the vessel wall.^108^ Vascular inflammation can suppress the differentiation of pre-adipocytes into adipocytes in PCAT, leading to increased lipolysis and reduced intracellular lipid droplet accumulation—processes mediated by pro-inflammatory cytokines such as IL-6, tumour necrosis factor-α, and interferon-γ.^109,110^

Several studies have observed transient increases in PCATa around culprit lesions in patients with coronary artery disease, suggesting that PCATa imaging might detect unstable plaques at an early stage.^83,84,111,112^ Longitudinal studies indicate that PCATa may predict the progression of individual plaques and changes in total coronary plaque burden.^79,113^ A meta-analysis highlighted a significant, though heterogeneous, difference in PCATa between stable and unstable coronary plaques.^114^

However, the ability of PCATa imaging to predict cardiovascular events has yielded mixed results. Some studies, such as the CRISP-CT and SCOT-HEART trials, demonstrated that high PCATa values are associated with MACE and that incorporating PCATa into traditional prognostic models enhances predictive accuracy.^82,84,115^ For instance, in the ORFAN study, which followed several thousand patients over nearly 8 years, there was a modest but significant association of increased PCATa values with increased risk of MACE after adjusting for other risk factors and plaque burden. Integration of PCATa, classical cardiovascular risk factors, and coronary plaque burden into an artificial intelligence (AI)-driven risk classification model led to improved cardiovascular risk prediction.^76^ Conversely, other studies have found that incorporating PCATa into risk assessments provided no incremental value to cardiac risk prediction in various at-risk populations,^77,78,81^ including one study with over 9 years of follow-up.^75^

CCTA imaging of PCAT inflammation holds promise for improving diagnosis and risk stratification in patients with coronary artery disease. However, substantial work is needed before PCAT imaging can be routinely used in clinical practice. Initial steps have been taken to standardize and validate optimal PCATa threshold values for each coronary artery, but PCATa values are influenced by factors such as technical platforms and patient anatomy, making current unvalidated thresholds insufficient for clinical use. Standardization of PCATa imaging protocols, including the anatomical sites assessed, is necessary. Moreover, most studies have been conducted in patient populations with clinical indications for CCTA. Evaluating the use of PCAT imaging in healthy populations will provide further insights into its potential benefits in primary prevention. As with the PET tracers discussed in this review, radiation exposure may hamper the widespread implementation of PCATa.

### Cardiovascular MRI

Cardiovascular magnetic resonance (CMR) imaging achieves spatial resolution on par with CCTA without exposing patients to ionizing radiation. Additionally, CMR offers superior soft-tissue contrast, potentially enabling detailed characterization of high-risk plaque structures; however, spatial and temporal resolution is often limited.^7^ However, data on CMR and its role in detecting vulnerable plaques are scarce and limited to the carotid arteries.^116^ Additionally, there are no data available on either the correlation between plaque features on CMR and cardiovascular outcomes or detection of inflammation on CMR beyond high-risk plaque features. This gap in evidence highlights the need for further studies to clarify CMR’s potential in identifying vulnerable plaques and improving prognostic accuracy over existing risk stratification methods.

## Future directions

Individual risk prediction and tailoring of therapeutic strategies to address inflammation in ASCVD is dependent on our understanding of associated markers. They aid in identifying those patients who have residual inflammatory risk and stand to gain the most from anti-inflammatory treatments. An ideal marker for this purpose should possess several key characteristics. First, the marker should have a high sensitivity, but more importantly, a high specificity to avoid unnecessary use of expensive therapies. Second, the marker or test should preferably be causally related to plaque inflammation and thus not a downstream surrogate of a general inflammatory process. Third, the marker should be reproducible, and inter-observer variability should be minimal. Fourth, such a marker should be affordable and able to be widely implemented in clinical practice.

With regard to these criteria, each method used to assess inflammation pertaining to ASCVD presents its own set of advantages and drawbacks (*Table 4*). The current standard of measuring plasma inflammation, such as hsCRP, is straightforward and, while effective on a population level, lacks precision on an individual patient level. PET imaging using ^18^F-FDG has shown good results for non-invasive visualization of vascular inflammation across multiple vascular regions but is challenged by limitations including limited spatial resolution and difficulties in imaging ^18^F-FDG uptake in coronary arteries, along with high costs and limited accessibility. ^18^F-NaF offers improved specificity over ^18^F-FDG in predicting events related to coronary inflammation, but larger-scale and prospective studies are required. ^68^Ga-DOTATATE is a tracer which could mitigate some of the limitations of ^18^F-FDG, including specificity and discriminatory capabilities; however, all PET-based methods are costly and have a relatively high radiation dose. CCTA imaging of high-risk plaque, most often inflammatory, is a widely employed method of stratifying cardiovascular risk. CCTA imaging of PCATa has emerged as a non-invasive, low-radiation biomarker with potential for wide availability and reasonable cost. It shows promise in imaging coronary vascular inflammation but needs further method standardization and threshold validation. While these methods for assessing inflammation in ASCVD have their unique advantages and limitations, no single marker or test currently meets all ideal criteria for precision, specificity, cost-effectiveness, and widespread clinical applicability.

## Multi-modal identification of inflammatory risk

Rather than relying solely on one mode of estimating inflammation, it is likely that clinicians will combine various modalities to predict ASCVD risk in the future. Integrating systemic biomarkers of inflammation with radiological markers of plaque inflammation—especially those that are high-throughput, low-cost, and reliable—could offer a personalized approach that goes beyond traditional clinical risk factors. Additionally, the incorporation of molecular biomarkers—such as proteomics and lipidomics, which have demonstrated robust predictive accuracy for cardiovascular events^117,118^—alongside imaging biomarkers may enhance risk stratification by providing a comprehensive view of both the molecular and structural aspects of ASCVD. With the advent of potent and selective anti-inflammatory interventions for treating residual inflammatory risk in atherosclerosis, these inflammation markers could serve not only as diagnostic tools but also as valid biomarkers of therapeutic response.

Emerging AI-driven models have the potential to further refine ASCVD risk assessment by integrating diverse data streams, including systemic, molecular, and imaging biomarkers. AI algorithms, particularly in machine learning, can analyse large datasets to identify patterns and associations that may not be apparent through traditional analysis, thereby supporting more precise inflammatory risk estimation.^119^ By applying AI to integrate imaging data with molecular and systemic biomarkers, clinicians can achieve a more personalized risk assessment, potentially improving patient outcomes.

Moving beyond inflammatory pathways, phenotyping other features of the arterial wall through advanced coronary artery imaging may enhance the understanding of an individual’s ASCVD risk. Incorporating genetic predisposition data, such as through polygenic risk scores—which compile the effects of numerous small genetic variations contributing to an individual’s overall susceptibility to ASCVD^120^—could add a further layer of precision. Combining detailed phenotypic observations with genetic predisposition and molecular data could offer a more comprehensive approach to assessing ASCVD risk. Integrating these data streams—inflammatory markers, phenotypic characteristics, genetic predisposition, and AI-driven analyses—could be a key in mapping the complex and dynamic factors that constitute an individual’s ASCVD profile and might lead to more targeted and effective prevention and treatment strategies.

## Conclusions

Several imaging techniques to capture the inflammatory component of atherosclerosis have been developed to date, allowing for improved phenotypic characterization of atherosclerotic lesions. These imaging modalities facilitate a transformation from a population-based approach to personalized inflammatory risk estimation. Integrating imaging of inflammation with molecular biomarkers, such as proteomics and lipidomics, might represent the best solution in current clinical practice for enhancing the accuracy of ASCVD risk prediction and enabling personalized therapeutic strategies. However, further studies are needed before a comprehensive inflammatory atherosclerosis imaging test—integrated with molecular biomarkers and potentially enhanced by AI analyses—can be readily implemented in clinical practice.

## Data Availability

No new data were generated or analysed in support of this research.
